# Editorial: Network Physiology, Insights in Dynamical Systems: 2021

**DOI:** 10.3389/fnetp.2022.961339

**Published:** 2022-07-15

**Authors:** Eckehard Schöll

**Affiliations:** ^1^ Institut für Theoretische Physik, Technische Universität Berlin, Berlin, Germany; ^2^ Potsdam Institute for Climate Impact Research, Potsdam, Germany; ^3^ Bernstein Center for Computational Neuroscience Berlin, Humboldt-Universität, Berlin, Germany

**Keywords:** dynamical systems, complex networks, network physiology, synchronization, coupled oscillators

The field of network physiology considers all aspects of collective dynamics of and on complex networks with application to functions and mechanisms in living systems (Ivanov, 2021; Schöll, 2021; Gosak et al., 2022). Complex networks are an ubiquitous paradigm in nature, with a wide field of applications ranging from physics, chemistry, biology, neuroscience, physiology, medicine to socio-economic systems. The human organism is an integrated network of organ systems, individual organs, cells, biomolecules, which all interact with each other on various levels. Rather than attempting to study the individual, isolated parts, the network perspective of dynamical systems focusses on the interaction between the different units which leads to the emergence of novel collective behavior not present in the isolated systems. Central to the physiological functioning are nonlinear, dynamic or adaptive biophysical and biochemical interactions, control mechanisms, communication and information exchange between cells and organs. This applies to the normal physiological state as well as to pathological states including diseases. Recent research on dynamical networks has revealed a plethora of collective dynamic phenomena. Synchronization is an important universal feature of the dynamics in networks of coupled nonlinear oscillators. Various synchronization patterns are known, like cluster synchronization where the network splits into groups of synchronous elements, or partial synchronization patterns such as chimera states where the system splits into coexisting domains of coherent (synchronized) and incoherent (desynchronized) states.

Phase transitions and critical phenomena in nonlinear dynamical systems far from thermodynamic equilibrium have been investigated since the 1970s and 1980s, and concepts from thermodynamics and statistical physics have been applied to describe self-organization, spatio-temporal pattern formation, phase coexistence, critical phenomena, and first and second order nonequilibrium phase transitions (Schlögl, 1972; Haken, 1978; Schöll, 1987). But only in recent times, phase transitions and critical phenomena have been studied in dynamical networks, where synchronization transitions may arise, giving birth to a plethora of partial synchronization patterns and complex collective behavior. These recent developments include connections of tipping transitions, explosive synchronization, nucleation, critical slowing down, critical correlations and fluctuations, critical exponents, etc. with nonequilibrium thermodynamics.

This Research Topic of Frontiers in Network Physiology – Networks of Dynamical Systems, is focused on new insights, novel developments, current challenges, latest discoveries, recent advances and future perspectives in the field. It features contributions from leading experts that describe the state of the art, outlining recent developments and major accomplishments that have been achieved and that need to occur to move the field forward. They deal with healthy as well as pathological states in brain networks, in general multistable networks, and in physiological networks describing the interaction of organs and the immune system, and are linked up with data-driven network approaches.

In the first contribution by Jifan Shi et al. it is suggested that criticality plays an important role in the healthy brain. The brain exhibits remarkable robustness under various types of noise and flexibility under various environments. However, how the brain works is still a mystery. The authors support the critical brain hypothesis by a dynamical network analysis using an electroencephalography dataset obtained from patients with psychotic disorders, ultra-high risk individuals, and healthy controls. The authors show that the brain of healthy controls remains around a critical state, whereas that of patients with psychotic disorders falls into more stable states. The brain of ultra-high risk individuals is similar to that of psychotic disorders in terms of entropy, but is analogous to that of healthy controls in causality patterns. These results not only provide evidence for the criticality of the normal brain but also highlight the practicability of using an analytic biophysical tool to study the dynamical properties of mental diseases.

Tobias Fischer et al. propose a data-driven estimation of resilience in multistable networked dynamical systems as a versatile testbed for normal and aberrant states. Estimating resilience of adaptive, networked dynamical systems remains a challenge for the analysis of empirical data, such as for example time series of physiological observables of the human organism. Resilience refers to the ability to absorb disturbances and still retain essentially the same functioning. The authors develop a data-driven approach and test it with a network of networks of diffusively coupled FitzHugh–Nagumo oscillators by modifying resilience in a controlled manner. For this purpose they simulate a multistable system with a number of system states and with self-induced switching between them. The testbed also enables generation of multivariate time series of system observables to evaluate the suitability of data-driven estimators of resilience.

M. Madadi Asl et al. consider Parkinson’s disease as a multi-systemic neurodegenerative brain disorder. Motor symptoms of Parkinson’s disease are linked to significant dopamine loss followed by basal ganglia circuit dysfunction. Increasing experimental and computational evidence indicates that synaptic plasticity plays a key role in the emergence of Parkinson’s disease-related pathological changes. Spike-timing-dependent plasticity (STDP) mediated by dopamine provides a mechanistic model for synaptic plasticity to modify synaptic connections within the basal ganglia according to the neuronal activity. This paper reviews experimental pre-clinical, clinical, and computational findings on the modulatory effect of dopamine on STDP as well as other plasticity mechanisms and discusses their potential role in Parkinson’s disease pathophysiology from the viewpoint of network dynamics. This review may provide further insights into the abnormal structure-function relationship within the basal ganglia contributing to the emergence of pathological states in Parkinson’s disease. Specifically, this review is intended to provide detailed information for the development of computational network models for Parkinson’s disease, serving as testbeds for the development and optimization of invasive and non-invasive brain stimulation techniques. Computationally derived hypotheses may accelerate the development of therapeutic stimulation techniques and potentially reduce the number of related animal experiments.

The last paper by Rico Berner et al. presents a novel approach to modeling healthy and pathological states in a functional physiological network of organs and the immune system. In this work, they propose a dynamical systems perspective on the modeling of sepsis and its organ-damaging consequences. They develop a functional two-layer network model for sepsis based upon the interaction of parenchymal cells and immune cells via cytokines, and the coevolutionary dynamics of parenchymal, immune cells, and cytokines. By means of the simple paradigmatic model of adaptively coupled phase oscillators, the emergence of organ threatening interactions between the dysregulated immune system and the parenchyma is analyzed. It is demonstrated that the complex cellular cooperation between parenchyma and stroma (immune layer) either in the physiological or in the pathological case can be related to dynamical patterns of the network, i.e., either a healthy frequency synchronized state, or a pathological multifrequency cluster state. Thus insight is provided into the complex stabilizing and destabilizing interplay of parenchyma and stroma by determining critical interaction parameters. Moreover, the simulated probability for the emergence of pathological states is compared with empirical data for the hospitalization incidence of sepsis in Germany as a function of age.
